# Circadian Network Interactions with Jasmonate Signaling and Defense

**DOI:** 10.3390/plants8080252

**Published:** 2019-07-28

**Authors:** Bryan Thines, Emily V. Parlan, Elena C. Fulton

**Affiliations:** Biology Department, University of Puget Sound, 1500 North Warner St., Tacoma, WA 98416, USA

**Keywords:** jasmonic acid, defense, circadian clock, signaling

## Abstract

Plants experience specific stresses at particular, but predictable, times of the day. The circadian clock is a molecular oscillator that increases plant survival by timing internal processes to optimally match these environmental challenges. Clock regulation of jasmonic acid (JA) action is important for effective defenses against fungal pathogens and generalist herbivores in multiple plant species. Endogenous JA levels are rhythmic and under clock control with peak JA abundance during the day, a time when plants are more likely to experience certain types of biotic stresses. The expression of many JA biosynthesis, signaling, and response genes is transcriptionally controlled by the clock and timed through direct connections with core clock proteins. For example, the promoter of Arabidopsis transcription factor MYC2, a master regulator for JA signaling, is directly bound by the clock evening complex (EC) to negatively affect JA processes, including leaf senescence, at the end of the day. Also, tobacco ZEITLUPE, a circadian photoreceptor, binds directly to JAZ proteins and stimulates their degradation with resulting effects on JA root-based defenses. Collectively, a model where JA processes are embedded within the circadian network at multiple levels is emerging, and these connections to the circadian network suggest multiple avenues for future research.

## 1. The Importance of Circadian Rhythms

The daily rotation of planet Earth and resulting day/night cycles affect nearly every location on the terrestrial surface. To match these cycles, organisms have an endogenous timekeeping mechanism called the circadian clock that confers 24-h rhythmicity to life processes. Clocks enable anticipation of daily changes in environmental conditions, match and coordinate internal processes with the external environment, and allow for efficient allocation of cellular resources [1,2]. For plants, important external conditions might occur with certainty, such as dawn or day length, and these events can help predict more variable environmental challenges, such as excessive temperatures or the presence of day-active pests. Clock-regulated rhythmic outputs in plants that increase preparedness for these correlative abiotic and biotic stresses often occur through regulation of hormones, such as jasmonic acid (JA) [3,4,5]. Recent work shows multiple molecular mechanisms by which the clock directly controls important aspects of JA action in plant growth and defense.

## 2. Plant Clock Mechanics: Genes and Proteins 

**Figure 1:**
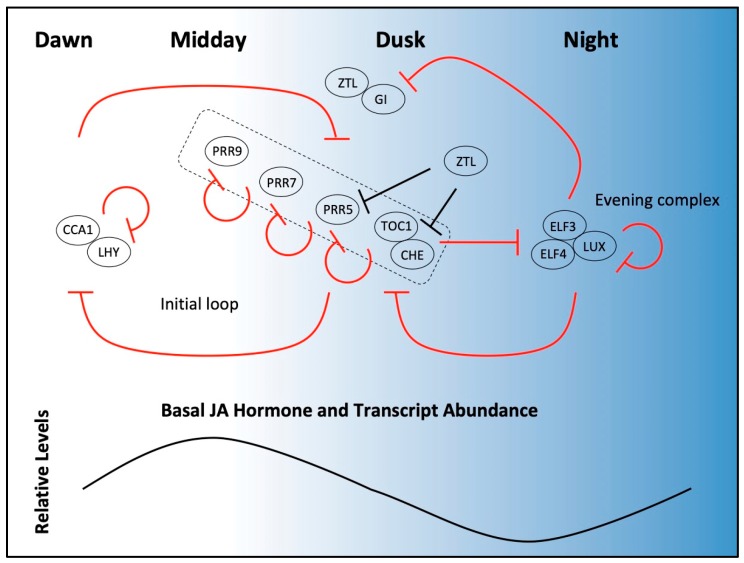
Progression of a simplified oscillator and JA processes over the 24-h day. Core oscillator components that are directly relevant to JA processes are shown (top) where proteins (ovals) appear at the time of their peak activity between dawn (left) and night (right). Transcriptional regulation events are depicted by red lines and post-translational regulation events are shown with black lines. The clock proteins shown at each phase, in general, suppress activities at other parts of the day. Morning proteins (CCA1/LHY) repress *PRR* genes, including *TOC1*, and themselves. PRR activity (grouped in box) is sequential throughout the day; PRRs repress previous *PRR* genes and *CCA1/LHY*. The evening complex (EC) represses *TOC1*, *GI*, and itself. F-box protein ZTL is stabilized by GI in blue light, but targets (without GI) PRR5, TOC1, and CHE for ubiquitylation and degradation in the dark. Proteins depicted here are relevant to the discussion of JA processes; other clock proteins exist with both repression and activation roles. JA biosynthesis genes, JA levels, and many JA response genes cycle over the course of 24 h and thus time many aspects of JA action to midday (bottom).

The classic view of the circadian system consists of three parts: (1) input signals from the environment (i.e., light and temperature) that give time-of-day cues and set the clock, (2) a core molecular oscillator that keeps 24-h time, and (3) rhythmic physiological outputs that are often manifested through cyclic gene expression programs [6,7]. Current views of the circadian system, however, acknowledge that different parts of this overall model (i.e., outputs) can influence other segments (i.e., inputs) in numerous ways leading to a robustly oscillating cellular network that is not necessarily unidirectional. The naturally occurring dark to light transition at dawn is important for clock setting, or *entrainment* [8]. Light is perceived by at least ten different photoreceptors that sense far-red, red, blue, and UV light [9]. Many details are known regarding the individual roles that various photoreceptors play in clock entrainment and progression, but more remains to be discovered about how this information is mechanistically coupled to the core oscillator [9]. Thermocycles also contribute to clock entrainment, with warmer temperatures acting as a daytime cue, although very little is understood regarding how temperature information is perceived and then coupled with the core oscillator [10,11]. The core oscillator consists of multiple integrated transcription–translation feedback loops (Figure 1). Clock genes within these loops are usually rhythmically expressed with peak transcript abundance timed, or *phased*, to a particular part of the day when their action is required for clock function [12,13]. Targeted protein degradation by the ubiquitin 26S proteasome system (UPS) is also prevalent throughout the clock, and this mechanism tunes the abundances of many clock proteins so that they also robustly cycle over a 24-h time course [14,15,16].

In plants, an initial loop includes the *CIRCADIAN CLOCK ASSOCIATED1* (*CCA1*) and *LATE ELONGATED HYPOCOTYL* (*LHY*) genes, which are rhythmically expressed with peak transcript levels at dawn (Figure 1) [13,17]. CCA1 and LHY are single MYB-domain transcriptional repressors that, during dawn hours, bind a DNA motif called the evening element (EE) found in promoters of many target genes that have evening-phased expression [18,19,20]. The EE is present in the promoter region of a *PSEUDO-RESPONSE REGULATOR* (*PRR*) family gene *TIMING OF CAB1* (*TOC1;* also known as *PRR1*) and helps limit *TOC1* expression to the later part of the day [18]. TOC1 is a DNA-binding transcriptional repressor which, along with CCA1 HIKING EXPEDITION (CHE), defines the evening part of this initial loop by directly repressing morning genes in the later parts of the day, including *CCA1* [12,20,21]. In addition to TOC1, four additional PRR proteins (PRR9, 7, 5 and 3) are sequentially expressed repressors that collectively restrict *CCA1/LHY* expression to dawn [22,23]. Each PRR represses the preceding *PRR*, in addition to other genes [18,19]. Thus, CCA1/LHY, TOC1, and additional PRRs have mutually repressive functions that limit the action of other clock genes to separate parts of the day.

Nighttime repression activity in the clock is provided by EARLY FLOWERING 3 (ELF3), EARLY FLOWERING (ELF4), and LUX ARRHYTHMO (LUX), each of which has oscillating gene expression and peak protein abundance at dusk [24,25,26]. ELF3, ELF4, and LUX associate to form a nuclear-localized ternary complex called the evening complex (EC) [27]. ELF3 and ELF4 have no domains of known function [24,25]. However, LUX is a GARP transcription factor with a single MYB domain that binds to LUX Binding Sites (LBS) and recruits the EC to target gene promoters, leading to their direct repression [26,28]. The EC directly represses other clock genes *TOC1*, *LUX*, *GIGANTEA* (*GI*), and *PRRs 7* and *9* [27,29,30]. The repression of these *PRRs* by the EC leads to elevated expression of *CCA1*/*LHY* as morning approaches [30]. The EC also directly represses important growth output genes, such as *PHYTOCHROME INTERACTING FACTOR*s (*PIFs*) at appropriate times of the day [27].

Protein abundance profiles of many clock proteins are tuned by selective protein degradation, where targets are identified by SCF-type E3 ubiquitin ligases [15,16,31,32]. The F-box protein ZEITLUPE (ZTL) is an SCF complex substrate adapter that recruits TOC1, PRR5, and CHE for ubiquitylation and subsequent degradation in the dark part of the daily cycle [15,16,31,32]. ZTL has an N-terminal LOV (Light, Oxygen, or Voltage) domain and is a blue-light photoreceptor, where perception of blue light alters binding affinities [32]. GIGANTEA (GI) interacts with the LOV domain of ZTL under blue light, which stabilizes ZTL and is essential for cyclic ZTL protein abundance that peaks at dusk [32]. ZTL family members, FLAVIN BINDING KELCH REPEAT F-BOX1 (FKF1) and LOV KELCH PROTEIN 2 (LKP2), also contribute to control the pace and robustness of the circadian clock through the regulation of TOC1 and PRR5 protein stability [16,33]. 

Over the years, other factors have been identified that are critical to clock function. TIME FOR COFFEE (TIC) is a clock regulator required for maintaining both period and amplitude of circadian rhythms [34,35]. TIC is nuclear localized, but it has no readily identifiable domains and its exact biochemical activity is unknown [34,35]. Additional activators and repressors occupy roles within the transcription–translation loops and/or connect signals into the clock [36,37,38]. Chromatin modification, transcript splicing dynamics, and additional post-translational mechanisms all finely tune rhythms and contribute to a robustly oscillating circadian network that appropriately times biological processes to the most beneficial times of the day [39,40]. 

## 3. Clock Phasing of JA Biosynthesis and Defense

JA is a well-established plant stress hormone required for inducible defenses against herbivore pests and necrotrophic pathogens [41,42], and it has important roles in growth and developmental processes [43,44]. Nearly every aspect of JA action, including biosynthesis, signaling, and downstream gene expression, is influenced by the circadian clock, which underscores the importance of biological timekeeping in plant growth and defense. While JA-regulated defenses are highly induced in response to pest or pathogen attack [41], these defenses can also occur at a lower, uninduced basal state that has significant effects [45]. This basal defense status likely minimizes costs incurred by defense pathway full activation, but still allows for initial readiness if the plant happens to be attacked. An energetic cost-saving measure is to anticipate the timing of potential threats and to phase JA basal defenses to the particular time of the day when they are most likely to be needed [5,18,45,46]. This increased readiness conferred by the clock in the case of biotic challenge gives plants a competitive advantage and results in increased survival [45,46,47]. 

The clock regulates defense against *Trichoplusia ni* (cabbage looper), a generalist herbivore that feeds on plants during the day and has maximal feeding activity just prior to dusk [45]. Arabidopsis wild type plants with clocks entrained in-phase with those of *T. ni* caterpillars and then released into constant conditions for herbivore challenge experience significantly less tissue damage, and *T. ni* loopers gain less weight than plants entrained out-of-phase. This illustrates an important hallmark of circadian processes in that they persist with rhythmicity under constant external conditions after clock entrainment to day/night cycles under driven conditions. Furthermore, mutants or transgenic plants devoid of oscillator function can help reveal the circadian nature of particular processes because events that are normally cyclic become arrhythmic after release from driven conditions into constant conditions. The enhanced resistance to *T. ni* in Arabidopsis plants with matched entrainment conditions is lost when the clock is rendered nonfunctional, such as in the *lux* mutant or plants overexpressing *CCA1*, indicating that effective defenses require circadian control [45,46]. The clock advantage is also lost in JA biosynthesis mutants *aos* and *jar1*, which lack active jasmonates [48,49,50], with these plants being equally susceptible whether entrained in-phase or out-of-phase with *T. ni* caterpillars [45]. JA levels are rhythmic with peak hormone abundance at midday [45]. Some genes encoding enzymes in JA biosynthesis are under clock control and cycle, which provides at least one means by which basal JA levels cycle [4,5]. In addition to synthesis, rhythmic removal of active JAs is likely important because: (1) the JA signal is antagonistic to different defenses used during different parts of the day (see below) [51], and (2) chronic induction of unneeded JA-mediated defenses, even at basal levels, would likely result in lost growth opportunities [52,53]. Multiple possible metabolic fates of JAs exist, some of which lead to inactivation [42], and the expression of at least one JA metabolizing enzyme, sulfotransferase ST2A, is influenced by the clock [54]. Finally, plants entrained while concurrently incubated with *T. ni* have elevated JA levels, though levels are still rhythmic, indicating that clock-controlled and insect-induced JAs are additive [45]. Interestingly, the uninduced levels of salicylic acid (SA), a hormone that regulates responses to biotrophic pathogens and has an antagonistic role to JA [55,56], are also rhythmic but phased to the nighttime [45]. Therefore, the clock acts to coordinate different defense programs controlled by these two opposing hormones (see Perspectives below).

## 4. JA Gene Suites Are Phased and Gated for Appropriate Time-of-Day Responses

Over 30% of the Arabidopsis transcriptome is clock-regulated and, within this cycling group, genes involved in hormone signaling and stress response pathways are overrepresented [4]. Furthermore, gene sets that are both induced and repressed by JA are significantly enriched for clock regulation and have rhythmic expression profiles [4,5]. Many defense genes such as the MYB-like transcription factor *PRODUCTION OF ANTHOCYANIN PIGMENT 1* (*PAP1*) and the JA biosynthesis gene *ALLENE OXIDE CYCLASE 1* (*AOC1*) have increasing expression throughout the day in uninduced conditions, a possible consequence of the JA hormone rhythms that coincide with potential exposure to day-active herbivores (see above; Figure 1) [4].

In addition to controlling cyclic basal gene expression levels, the clock can also regulate the responsiveness of a gene, which is referred to as *gating*, and some genes are more responsive to stimuli at one time of the day versus another [57]. JA is critical to defense against *Botrytis cinerea*, a necrotrophic fungal pathogen with a broad host range, and the clock gates an increased response and enhanced resistance in the earlier part of the day near dawn in Arabidopsis [58,59]. In Arabidopsis plants with entrained clocks, this time-of-day enhanced resistance to *B. cinerea* persists in constant conditions but is lost in *elf3-1* and *cca1 lhy* clock mutants [58]. This is consistent with *B. cinerea* fungal spore release being diurnally regulated, with spores most numerous in the morning and early afternoon [59]. Large-scale transcriptional reprogramming is a critical host response following detection of *B. cinerea* in Arabidopsis [58]. Hundreds of genes are more highly induced when *B. cinerea* inoculation occurs at dawn compared to dusk, and these morning high response genes are significantly enriched in JA genes that are involved in JA metabolism, signaling, and response [58]. By regulating (via gating and phasing) both basal and inducible JA gene expression, the clock facilitates efficient defense programming, which is primed to match pest and pathogen daytime-dependent activities. 

## 5. Core Clock Transcription Factors Directly Regulate JA Genes

In addition to gene expression programs being rhythmically activated as a consequence of cyclic JA levels and signaling, some JA genes are directly regulated by DNA-binding core clock proteins. Because many clock proteins have rhythmic accumulation profiles, their presence and activities provide a direct connection between the clock and temporal expression of output JA genes [12,13,19,28]. Nearly one hundred genes encoding transcription factors were identified in the group of genes described above that highly respond to *B. cinerea* at dawn [58]. About half of these transcription factor genes cycle, and this rhythmic group is enriched in genes that are direct targets TOC1, PRR5, PRR7, which are core clock DNA-binding transcription factors that repress genes late in the day (see above; Figure 1) [58]. 

Some JA biosynthesis and response genes are directly regulated by other clock proteins, such as CCA1 or LUX [19,28,60]. The EC (via LUX) binds LBS motifs and represses associated morning/early day genes at dusk and into the night [26]. The LBS is present in the promoter regions of many genes encoding core jasmonate signaling components *COI1*, *JAZ1*, *JAZ5*, *MYC2*, *MYC3*, and *MYC4* [28,60]. LUX directly interacts with regulatory regions of at least *MYC2* and *JAZ5*, and associated LUX repression effects have been validated in the case of *MYC2* [28,60]. Consistent with the direct transcriptional repression role of the EC, over 70% of the nearly thousand genes mis-expressed in *lux* mutants at dusk are up-regulated, and these mis-expressed genes are significantly enriched in JA signaling and defense genes, as well as genes related to senescence [60]. Mutant *lux* plants, like *elf3* mutants, exhibit more severe lesions on leaves treated with *B. cinerea* [28].

End-of-day repression of JA signaling genes by the EC is important for age-related regulation of senescence, in addition to defense responses (see below) [60]. Arabidopsis EC mutants *elf3*, *elf4*, and *lux* all have common early leaf senescence phenotypes that are accelerated by JA more than in wild type, while in contrast *ELF3* over-expressing plants have a staying-green phenotype [60]. Some MYC transcription factors, in particular MYC2, are master regulators of JA signaling and promote JA-induced senescence [61]. Mutations in *MYC2*, *MYC3*, and *MYC4* genes suppress the accelerated JA senescence in *elf3* and *lux*, indicating that the EC impacts JA senescence processes, at least in part, by repressing *MYC* function [60]. Interestingly, the clock runs faster in older leaves [62], and this shorter period, possibly coupled with seasonal day-length changes, may influence EC regulation of senescence through LUX binding of JA- and senescence-related target genes [63].

## 6. The COI1-JAZ-MYC Core JA Signaling Module Is a Target for Gating and Phasing

**Figure 2:**
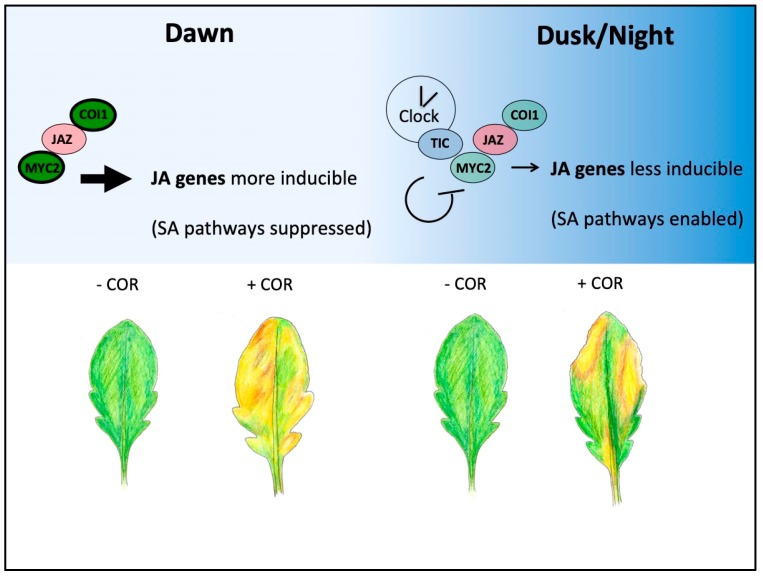
Gating of JA responses through gene expression control and by TIC. COI1-JAZ-MYC signaling complex positive components (green) and negative components (red) are shown in the morning (left) or evening (right); more intense coloring and darker border indicate higher protein abundance. Throughout the night and at dawn, the clock protein TIC reduces MYC2 protein abundance likely through direct interaction, and it therefore restricts MYC2-dependent actions (right side) [76]. Positive factors of the COI1-JAZ-MYC2 complex (COI1, MYC2) are more abundant at dawn and during the daytime due to transcriptional or post-translational control, and this is one mechanism that gates JA responsiveness to this time of the day. JA early genes (i.e., *JAZ5*) are most inducible by JA treatment at dawn [76]. The JA mimic coronatine (COR) produced by *Pseudomonas syringae* (*Pst* DC3000) hyper-activates the JA pathway and suppresses SA-dependent and independent host defenses against biotrophic pathogens [77,78]. Treatment of plants at dawn with *Pst* DC3000 results in leaves that support more pathogen proliferation compared to treatment at dusk, although clock-timing of stomata closure that is independent of JA signaling could play an important role too (bottom) [76,79].

Clock action on the COI1-JAZ-MYC core signaling module is another direct way by which JA processes are temporally controlled (Figure 2). CORONATINE INSENSITIVE1 (COI1) is an F-box protein and substrate adapter in the SCF^COI1^ complex that targets JASMONATE-ZIM-DOMAIN (JAZ) proteins for ubiquitylation and subsequent degradation in JA signaling [64,65,66,67]. COI1 and JAZ proteins associate to form a complete receptor complex in the presence of active JAs (i.e., JA-Ile, JA-Leu), where the JA molecule acts as molecular glue to stabilize the association [68]. JAZ proteins are repressors in JA signaling that bind to and suppress the function of a family of DNA-binding MYC transcription factors and, directly or indirectly, recruit additional co-repressors in the TOPLESS family [69,70,71]. Arabidopsis MYC2, MYC3, MYC4, and MYC5 are group IIIe bHLH transcription factors and master regulators of JA-regulated gene expression [72,73]. MYC proteins can homo- and heterodimerize and have both overlapping and distinct roles in regulating JA responses [71,72,73]. MYC2 is the best-studied of the family, and it binds to G-boxes and related sequences in the promoters of JA early response genes, including members of the *JAZ* gene family [74].

Controlling the COI1-JAZ-MYC2 module over a 24-h period provides opportunities to not only phase JA gene peak expression to certain parts of the day, but also to gate the magnitude of response to a particular time window. Shifting the balance in relative nuclear abundances between positive (COI1, MYC proteins) and negative (JAZ proteins) acting components can alter the overall activity and sensitivity of this COI1-JAZ-MYC complex to achieve these effects (Figure 2, top) [75]. Arabidopsis *COI1* and *MYC2* genes are under diurnal and clock control, respectively: *COI1* expression peaks at dawn, a time when plants respond maximally to JA, while *MYC2* transcript is most abundant near subjective dusk [76]. However, in *MYC2* over-expressing (*MYC2ox*) Arabidopsis plants with constant transcript abundance, MYC2 protein levels are also rhythmic and peak around midday in both diurnal and constant conditions, with levels dropping off significantly after dark [76]. Thus, both of the positive acting components of core JA signaling appear to have elevated activities in the daytime due to transcriptional and post-translational mechanisms.

## 7. The Clock Gates JA Signaling by TIC-Mediated Changes in MYC2 Protein Abundance 

The clock protein TIC is an important means by which the clock restricts MYC2 activity in both growth and defense processes (Figure 2). Although the biochemical function of TIC is unknown, it physically interacts with MYC2 in the nucleus [76]. MYC2 protein levels remain cyclic in *tic* mutants and peak at midday, indicating that TIC does not influence MYC2 phasing; however, MYC2 levels are higher at all times of the day in *tic* plants, with the most noticeable increases at night and at dawn [76]. Therefore, TIC acts to reduce MYC2 levels and JA responsiveness. Arabidopsis *myc2* seedlings are resistant to JA in root growth assays and *tic* seedlings have enhanced sensitivity, while this sensitivity is completely lost in the *tic myc2* double mutant [76]. Leaves from Arabidopsis *myc2* mutant plants are more resistant to *Pseudomonas syringae* (*Pst* DC3000) infection and those from *tic* are more sensitive compared to wild type, while much of this sensitivity is lost in leaves from the *tic myc2* double mutant [76]. Finally, some MYC2-dependent JA genes are hyperactivated in *tic* mutants [76]. Collectively, these genetic analyses show that TIC works in the same pathway as MYC2, at least in many important aspects, to restrict MYC2 function in a time-of-day dependent manner. 

## 8. The Clock Acts through JAZ Repressors to Control JA Signaling

**Figure 3:**
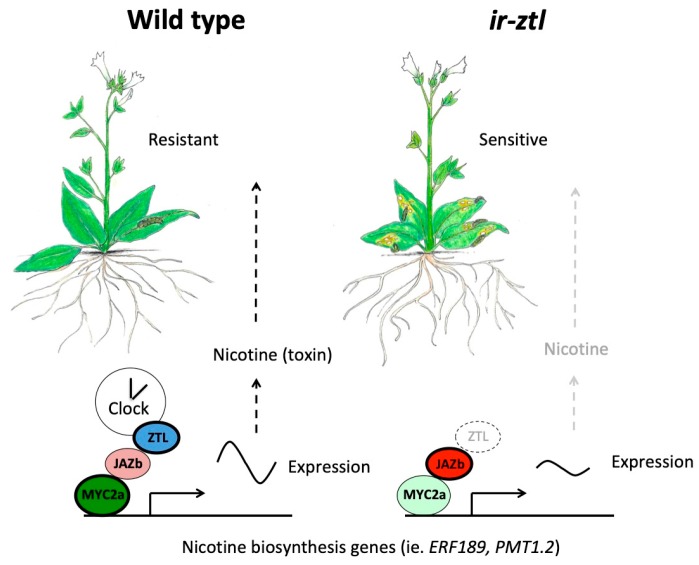
The clock protein ZTL directly regulates JA signaling and nicotine biosynthesis in *Nicotiana attenuata* roots. Nicotine is a neurotoxin produced in *Nicotiana* species that is synthesized in roots and transported to shoots where it accumulates and acts as a feeding deterrent (left side) [81]. Some underlying genes in nicotine biosynthesis are rhythmically expressed and/or are directly controlled by master regulator MYC2 homologs in *Nicotiana* species [80,82]. *N. attenuata* plants with reduced abundance of clock protein ZTL through expression of an inverted repeat construct (referred to *ir-ztl*) have defective rhythms, due to the role of ZTL in the clock [80,83] (right side). Both basal and induced JA levels are not substantially different between wild type and *ir-ztl* plants [80]. However, plants with reduced ZTL expression have reduced nicotine levels and diminished resistance to the generalist herbivore *Spodoptera littoralis* (Egyptian cotton leafworm) as measured by worm biomass, a defect that is rescued by exogenous application of nicotine [80]. Nicotine biosynthesis genes in *ir-ztl* plants are expressed at lower levels and with dampened rhythms [80]. ZTL physically interacts with the JAZb repressor protein with a resulting decrease in JAZb abundance (left) [80]; more intense coloring and darker border indicate higher protein abundance. Loss of ZTL function in *ir-ztl* plants likely increases JAZ presence and decreases *N. attenuata* MYC2a action in nicotine biosynthesis, among other processes (right). ZTL therefore contributes to herbivore resistance in *Nicotiana* by enabling MYC2 gene expression capabilities through JAZ destabilization that may depend on the time of day.

The clock also influences JA signaling by altering the restraint imposed by JAZ proteins. In *Nicotiana attenuata* (wild tobacco), the clock F-box protein ZTL affects JA signaling through physical interaction with multiple members of the JAZ protein family (Figure 3) [80]. However, the binding mechanism between ZTL and JAZ proteins appears to be different than that of COI1 and JAZ. JAZ proteins interact with COI1 via a Jas domain located near their C-termini, leading to JAZ ubiquitylation and degradation, but the more centrally located TIFY region of JAZ proteins is used to interact with the LOV domain in ZTL [77,80]. Furthermore, while a JA ligand (i.e., coronatine or JA-Ile) is required for COI1 interaction with JAZ proteins, no such requirement exists in the case of ZTL-JAZ interaction [80]. In vitro protein degradation assays with plant extracts show that *N. attenuata* ZTL has a destabilizing effect on JAZb, suggesting that ZTL might act to limit JAZ protein function at some part of the day [80]. At this point, however, it is unknown whether JAZ proteins are directly recruited by ZTL for ubiquitylation in a manner similar to TOC1 and PRR5 (see above), or whether there are time-of-day or blue-light dependencies in ZTL-stimulated JAZ degradation in plants. For example, ZTL interacts with GI in blue-light (day) but interacts with and targets TOC1 and PRR5 for degradation at night [31,32].

In addition, JAZ proteins appear important to mediating the clock gating effects leading to enhanced *Botrytis cinerea* responses at dawn, but increased susceptibility at dusk (see above) [58]. Arabidopsis plants with the *jaz6* mutation, but not *jaz5*, *7*, or *10* lesions, lose the enhanced susceptibility at subjective night and there are no longer significant time of day differences in lesion size [58]. This indicates that JAZ6 is employed by the clock to preferentially direct the *B. cinerea* response to morning and daytime hours, and it is an interesting example of subfunctionalization harnessed by the clock within the *JAZ* gene family. Collectively then, the clock alters JAZ function, as well as MYC2 function, to phase and gate action and sensitivity of the COI1-JAZ-MYC2 complex to particular windows within the day. 

## 9. Perspectives

Many JA-regulated biological processes are subject to circadian control and preferentially occur during particular times of the day. This optimized timing reflects the most beneficial allocation of limited energy and resources possessed by plants in the face of persistent environmental challenges and constraints. Only in the past few years have actual mechanistic connections between the clock and JA become known, which we have discussed here. Through specific protein–protein and protein–DNA interactions, the clock tunes all aspects of JA action, including biosynthesis, signaling, and expression of JA gene response programs. 

The array of bioactive molecules collectively referred to as “jasmonates” has long intrigued plant biologists intent on understanding their distinct and overlapping functions in various plant processes [84,85]. Whether the clock preferentially influences some of these jasmonates or their fates over others is unknown, and future work could investigate differential clock effects on distinct chemicals within this group. Clock regulatory activity over opposing JA and SA hormone pathways, in addition to other defenses, exemplifies the notion that the clock is a master regulator of cellular processes [28,45]. Very recent work has yielded mechanistic insights by which the clock directly regulates opposing pathways through transcriptional regulation; LUX binds regulatory regions of genes encoding central regulators of JA signaling *MYC2* and *JAZ5*, as well as genes that are central to the regulation of SA processes, such as *EDS1* [28,60]. This work continues to inform us that clock control of JA signaling is complexly woven into the regulation of other defense systems and should not be viewed in isolation [28,45]. Advantages afforded by high throughput sequencing approaches and other new technologies yield an unprecedented level of detail in this regard, and these types of approaches will likely provide important new connections as to how JA relates to other hormones, and also higher resolution understanding of these processes throughout the day.

JA also appears to have multiple emerging roles in abiotic stress responses and it will be interesting to examine how the clock integrates in these situations [86,87,88,89]. Importantly, a discussion of the active role that JA plays in defense and other stress responses cannot be separated from its broader effects on plant growth and developmental processes. It is now well recognized that activation of JA defense pathways restricts growth processes, and this hormone is thus a critical regulator in balancing these competing plant needs [53,90]. In addition to JA-inhibition of root growth and promotion of leaf senescence indicated above, JA has inhibitory effects on leaf expansion, hypocotyl growth, petal expansion, and flowering [52]. JA has essential roles in plant reproduction and fertility, and the clock could be an important factor in coordinating these reproductive processes with favorable external factors [43,44]. How the clock influences JA in this wide array of processes awaits investigation and future studies in this area could address questions relating to how plants balance growth and defense requirements over the course of the day. Optimal growth and defense strategies also impart a division of labor with certain processes occurring in particular tissues and organs. As described above, clock controlled regulation of nicotine biosynthesis in roots affects herbivory at the whole plant level [80]. How the core clock is coupled to specialized organ- and tissue-specific JA processes will likely be an interesting avenue of research, but whether this uniqueness arises either through exclusive downstream events from a universal clock or due to tissue-specific clocks is unknown [91]. 

Finally, circadian clock genes underlie desirable traits relating to growth and biomass, reproduction, and yield in present-day domesticated crops [92]. However, with an eye towards the future, global climate change is altering feeding and reproductive behaviors, geographical distributions, and migration patterns of many plant pests and pathogens, in addition to altering the severity of abiotic stresses (i.e., excessive temperature and drought) [93,94]. Therefore, the clock and JA interface will most certainly be important to understand toward future crop selection and improvement in this changing world.
